# Diagnostic accuracy of tomosynthesis-guided vacuum assisted breast biopsy of ultrasound occult lesions

**DOI:** 10.1038/s41598-020-80124-4

**Published:** 2021-01-08

**Authors:** Suhaila Bohan, Marlina Tanty Ramli Hamid, Wai Yee Chan, Anushya Vijayananthan, Norlisah Ramli, Shaleen Kaur, Kartini Rahmat

**Affiliations:** 1 Department of Biomedical Imaging, University of Malaya Research Imaging Centre, 50603 Kuala Lumpur, Malaysia; 2 Department of Radiology. Faculty of Medicine, University Teknologi MARA, Sungai Buloh Campus, Selangor, Malaysia

**Keywords:** Health care, Medical imaging

## Abstract

This study aims to evaluate the diagnostic accuracy of digital breast tomosynthesis-guided vacuum assisted breast biopsy (DBT-VABB) of screening detected suspicious mammographic abnormalities comprising of calcifications, asymmetric densities, architectural distortions and spiculated masses. In this institutionally approved study, a total of 170 (n = 170) DBT-VABB were performed, 153 (90%) were for calcifications, 8 (4.7%) for spiculated mass, 5 (2.9%) for asymmetric density and 4 (2.4%) for architectural distortion. All these lesions were not detected on the corresponding ultrasound. Histopathology results revealed 140 (82.4%) benign, 9 (5.3%) borderline and 21 (12.4%) malignant lesions. The total upgrade rate at surgery was 40% for atypical ductal hyperplasia and 5.9% for ductal carcinoma in-situ. 3.6% discordant benign lesions showed no upgrade. DBT-VABB showed 100% specificity, 91.3% sensitivity and 100% positive predictive value (PPV) for detecting malignant lesions. The negative predictive value (NPV) was 80%. 2 (1.2%) patients had mild complications and 1 (0.6%) had severe pain. Our study showed that DBT-VABB was a safe and reliable method, with high sensitivity, specificity, PPV and NPV in the diagnosis of non-palpable benign and malignant breast lesions. Our data also confirmed the accuracy of DBT-VABB in detecting malignant lesions and we suggest further surgical excision in borderline lesions for a more accurate diagnostic evaluation.

## Introduction

Breast cancer remains the leading cancer in Malaysian female and worldwide, with a total of 21,634 cases diagnosed from year 2012 to 2016 as compared with 18,206 in 2007 to 2011, based on the second 5 year report from Malaysian National Cancer Registry. It accounts for 34.1% of all cancer among Malaysian females with the age-standardised incidence rate (ASR) increased from 31.1 in previous report to 34.1 per 100,000 populations. The incidence is highest among Chinese followed by Indians and Malays^1^.

Early detection of breast cancer reduce morbidity, mortality and improve the survival rate of breast cancer patients^2–6^. Mammography and ultrasound (US) are widely used imaging modalities for breast screening whereas magnetic resonance imaging (MRI) breast is reserved for screening of high-risk patients. Breast cancer awareness campaign and advocates have lead to a surge in patients coming for screening and hence, increases the detection rates of various lesions. Screen detected lesions are mostly non-palpable and commonly manifest as calcifications + /- mass. Management of uncertain breasts calcifications are challenging, and histopathological (HPE) confirmation are often required.

Breast calcifications are one of the most common findings on mammography. Both benign and malignant lesions can produce mammographically suspicious calcifications and it is an important indicator in the early stage of breast cancer^7–9^. According to local cancer registry, 80% of ductal carcinoma in-situ (DCIS) cases are found during screening mammography without palpable lump and their earliest sign is microcalcifications^8,10^. Other less common mammographic abnormalities are masses, architectural distortion and asymmetries. Breast Imaging Reporting and Data System (BI-RADS) by American College of Radiology (ACR) guidelines 5th edition, 2013 helps to stratify the microcalcifications and aids in their subsequent management.

Stereotactic guidance percutaneous biopsy has widely replaced surgical excision for histologic verification of indeterminate or suspicious lesions detected only on mammogram^7,11–13^. Vacuum assisted breast biopsy (VABB) is a newer technique and is currently the preferred method over core needle biopsy (CNB) for diagnostic evaluation.VABB is a minimally invasive procedure that is able to obtain larger samples without repositioning or reinsertion. It has been demonstrated to be a safe alternative to open surgical biopsy and shows numerous advantages over surgical excision including desirable cosmetic results, fewer complications, less psychological stress, more cost-effective and less hospital stay^11,12^. It can be done either under stereotactic, ultrasound or MRI guidance.

Diagnostic underestimation of DCIS and borderline lesions, especially atypical ductal hyperplasia (ADH), are not uncommon in stereotactic guidance percutaneous biopsy. A wide range of percentages have been reported and the majority of published literature concluded that CNB has a higher rate of diagnostic underestimation compared to VABB^13–15^.

In Malaysia, DBT-guided stereotactic VABB (DBT-VABB) is not widely used for diagnostic purposes as it is not readily available in all hospitals. It is currently mostly being utilized in tertiary referral hospitals with breast surgery and oncology subspecialties. In this study, we present our experience of utilizing DBT-VABB and evaluate the diagnostic accuracy of this method in non-palpable mammographic breast lesions.

## Methodology

### Study design

This was a single-center retrospective cross-sectional study, involving all patients with BI-RADS category 4 and 5 mammographic lesions who underwent DBT-VABB from 1st March 2017 to 31st July 2019. These lesions were detected on routine screening mammogram, occult on US and include calcifications, asymmetric densities, spiculated masses and architectural distortions. The study was performed in adherence with the approved guidelines from Medical Ethics Committee of University Malaya Medical Centre (MECID No: 2018724–6517). All experimental protocols were approved by Medical Ethics Committee of University Malaya Medical Centre and all subjects provided written informed consent. Information on the location, morphology and distribution of lesions and patients’ clinical data including age, risk factor, number of samples, residual lesion, tissue marker insertion, complications, HPE findings and any subsequent surgery were collected. Pain scoring assessment post-VABB was also recorded. Patients with confirmed breast cancer on the contralateral breast were excluded.

### Equipment and technique

All patients were examined on DBT (Selenia Dimensin Hologic, Bedford, Massachusetts). Patients subsequently had supplementary ultrasound evaluation of the breasts and both axilla using diagnostic B-mode greyscale and colour, medical grade US system (Philips iU22; Philips Healthcare, Bothell, WA, USA) with a high frequency (i.e. 12.5 MHz) linear transducer probe. Ultrasound scans were performed by final year radiology trainee and the findings were verified by radiologists in-charge of the posting. DBT-VABB were performed by consultant radiologists with more than 10 years experience using the Mammotome system (Devicor Medical Products, Leica Biosystems). Few cases were done using EnCore Enspire system (Bard BD). 10G stereotactic biopsy needles were used for both systems.

The biopsy procedure was done in the upright position. Scout images were taken to confirm the presence of lesion within the field of projection and determined the puncture target. In patients with diffuse and regional calcifications, puncture target was made at the site with the densest distribution of  calcifications. Pre-fire and post-fire images were taken before DBT-VABB was performed. For calcifications, specimen radiograph was performed to confirm the presence of calcifications. Post-procedure mammogram was also performed to confirm on the complete or partial removal of lesion.

### Data collection and analysis

Data were retrieved from local picture archiving and communication system (PACS) and two readers (consensual method), blinded to the pathology results, categorised the findings according to ACR-BIRADS 5th edition (2013) mammogram lexicon; WYC and MTRH (with 5 and 10 years of mammography experience). HPE results were classified as benign, borderline or malignant (DCIS + invasive carcinoma). Final surgical pathology were also documented for patients who underwent subsequent surgical excision. Post-VABB complications were documented. Patients’ pain were scored using Numeric Rating Scale (0–10) and categorised as none (0), mild (1–3), moderate (4–6), severe (7–9) and worst pain (10).

### Statistical analysis

Statistical analysis was performed with a statistic software, using Statistical Package for the Social Sciences (SPSS) for Windows Version 23.0 (IBM corporation, Armonk, NY). For quantitative data, range and median were reported. Descriptive statistics were used and data were presented as frequency (*n*) and per cent distribution (%). Diagnostic underestimation was calculated by dividing the number of upgrade in surgical HPE by the number of ADH or DCIS in VABB, respectively. Differences between proportions were tested using a Pearson’s chi-square test. Fisher exact tests were used when any assumptions of Pearson’s chi-square test were violated. A p-value of < 0.05 was applied as a threshold for statistical differences.

**Figure 1:**
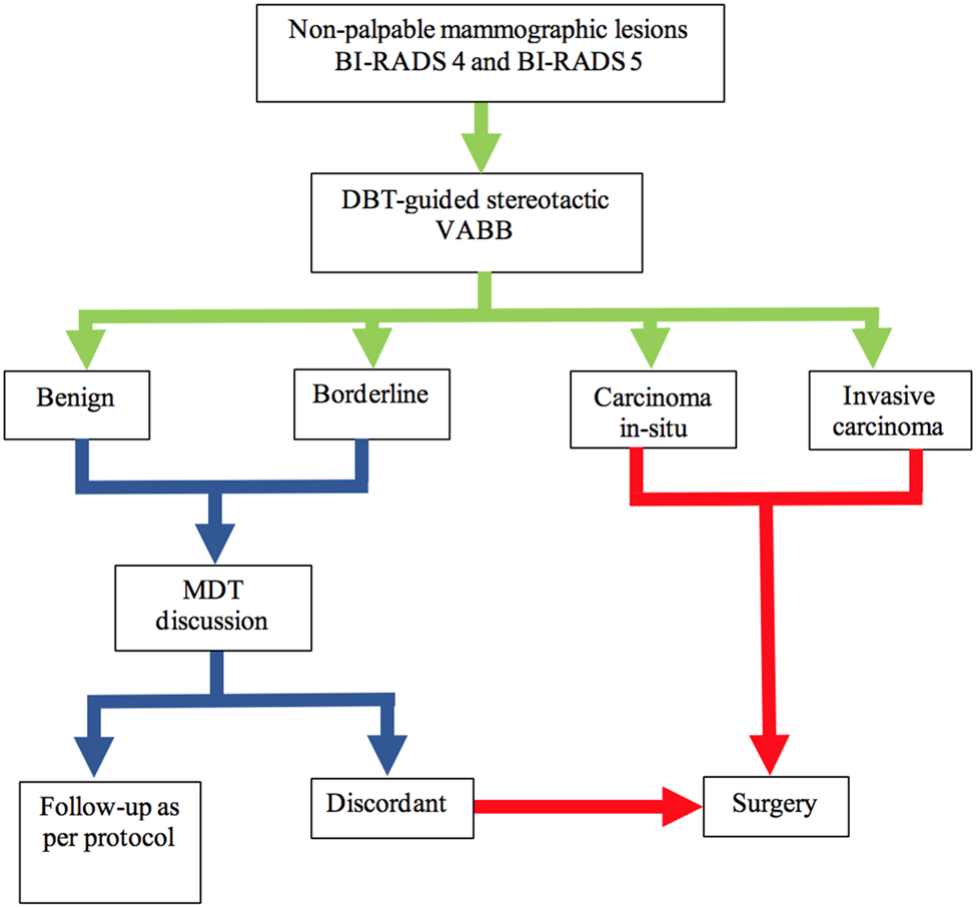
Flow chart of lesions and further management.

Figure 1 outline the flow of the lesion from the initial detection to surgical management or follow-up. All carcinoma in-situ and invasive cancer will proceed to surgery. All benign and borderline lesions will be discusssed in multidisciplinary team (MDT) meeting and radiology-pathology discordant lesions will proceed to surgery.

### Informed consent

Written informed consent was obtained from the patients.

## Results

### Study population and clinical data

There were a total of 170 (n = 170) patients recruited. The age ranged were between 34 to 77 years old, with a mean of 56.6 (SD 10.1) years. The most frequent age range was 50–59 (n = 55, 32.4%). 73 (42.9%) had previous history of breast cancer, 18 (10.6%) had a strong family history of breast cancer and 79 (46.5%) had no risk factor.

There was no preponderance of site for breast lesions with 69 lesions on the right and 101 lesions on the left. The majority of the lesions were seen in the upper outer quadrant, *n* = 92 (54.1%). 9 (42.9%) malignant lesions were seen in the right and 12 (57.1%) in the left breast. 60 (40.3%) benign lesions were seen in the right and 89 (59.7%) in the left breast.

### Mammographic findings

For BI-RADS breast density category, there were 9 (5.3%) patients in density category A, 58 (34.1%) in category B, 85 (50%) in category C and 18 (10.6%) in category D.

The majority of lesions were calcifications 153 (90%), followed by spiculated mass 8 (4.7%), asymmetric density 5 (2.9%) and architectural distortion 4 (2.4%).

For calcifications morphology, coarse heterogeneous type was the most commonly seen with 55 (35.9%) lesions, followed by fine pleomorphic in 52 (34.0%), amorphous in 38 (24.8%) and fine linear in 8 (5.2%). For distribution of calcifications, most were seen in the grouped category, 76 (49.7%) and the least were in the diffuse category, 13 (8.5%).

In the final BI-RADS assessment category, they were 103 (60.6%) BI-RADS 4a, 39 (22.9%) BI-RADS 4b, 25 (14.7%) BI-RADS 4c and 3 (1.8%) BI-RADS 5 lesions.

### Biopsy procedures

154 (90.6%) lesions were biopsied using Mammotome machine and 16 (9.4%) using Bard Encor machine. The number of specimens collected from the lesions during VABB ranged from 5 to 27 with a mean of 6.8 (SD2.6).

Tissue marker clip was inserted in all lesions with clip migration occurring in 11.8% of patients. The distance of clip migration from the biopsy site ranged from 0.4 cm to 4 cm.

Overall, only 2 patients developed a small haematoma confined to the biopsy site, measuring less than 2 cm. No severe complication was recorded.

For pain assessment, most patients (*n* = 94, 55.4%) only experienced mild pain. 1 (0.6%) patient had severe pain and this was associated with high number of specimens sampled, *n* = 24 and the resultant development of a small haematoma. These data were presented in Table 1.

**Table 1:** Demographic and lesion’s data.

General characteristics	Total cases (*n* = 170)
	*n* (%) / mean ± SD
**Age, years**	56.6 ± 10.14
**Risk factor**	
No Risk Factor	79 (46.5)
Previous Carcinoma	73 (42.9)
Strong Family History of Cancer	18 (10.6)
**Location in breasts**	
Right	69 (40.6)
Left	101(59.4)
**Location by quadrant**	
Upper outer (UOQ)	92 (54.1)
Upper inner (UIQ)	4 (2.4)
Central	54 (31.8)
Lower outer (LOQ)	12 (7.0)
Lower inner (LIQ)	8 (4.7)
**BI-RADS classification**	
4a	103 (60.6)
4b	39 (22.9)
4c	25 (14.7)
5	3 (1.76)
**Lesions morphology**	
Calcifications	153 (90)
Asymmetric Density	5 (2.9)
Architectural Distortion	4 (2.4)
Spiculated Mass	8 (4.7)
**Morphology of calcifications, n = 153**	
Coarse heterogenous	55 (35.9)
Amorphous	38 (24.8)
Fine pleomorphic	52 (34)
Fine linear	8 (5.2)
**Distribution of calcifications n = 153**	
Diffuse	13 (8.5)
Grouped	76 (49.7)
Regional	30 (19.6)
Linear	15 (9.8)
Segmental	19 (12.4)
**Marker clip insertion**	
Yes	170 (100)
**Marker clip migration**	
No	150 (88.2)
Yes	20 (11.8)
**Pain score**	
No pain	44 (25.9)
Mild pain	94 (55.3)
Moderate pain	31 (18.2)
Severe pain	1 (0.6)
**Complication**	
No	168 (98.8)
Yes	2 (1.2)

### VABB HPE

There were 149 (87.6%) benign and 21 (12.4%) malignant lesions. The most common benign lesions were fibrocystic disease, *n* = 59 (34.7%). Out of these benign lesions, there were 9 borderline lesions, majority being ADH (*n* = 7). For malignant lesions, they were 17 DCIS (10%) and 4 invasive carcinomas (2.4%). Detailed HPE data are shown in Table 2.

**Table 2:** HPE findings in 170 VABB lesions.

Benign	*n* = 14*9* % (87.6)	Malignant	*n* = 21 % (12.4)
**Benign**	140 (82.4)	**Invasive carcinoma**	4 (2.4)
Fibrocystic disease	59	Infiltrating ductal	3
Fibroadenoma	11	Invasive papillary	1
Usual ductal hyperplasia (UDH)	12	**DCIS**	17 (10.0)
Sclerosing adenosis	10	Low grade	5
Other benign lesions*	48	Intermediate grade	5
**Borderline**	9 (5.3)	High grade	7
Atypical ductal hyperplasia**	7		
Flat epithelial atypia**	1		
LCIS**	1		

*Other benign lesions include; benign breast tissue, mammary duct ectasia and no malignancy.

**Borderline lesions.

### Comparison of VABB HPE with mammographic findings

VABB HPE results in relation to mammographic findings were presented in Table 3. The majority of VABB patients were in BI-RADS 4a category. All BI-RADS 5 lesions were malignant. Fisher Exact test demonstrated statistically significant differences in VABB HPE among BI-RADS category (p < 0.01). This study demonstrated that positive predictive value (PPV) in detecting malignant lesion increases in tandem with BI-RADS category.

**Table 3:** VABB HPE with mammographic findings including BI-RADS category, lesions morphology and distribution and morphology of calcifications.

Mammographic findings	VABB HPE, *n*(%)				PPV %
	Benign		Malignant		
	**Benign**	Borderline	DCIS	Invasive	
**BI-RADS (n = 170)**					
4a (*n* = 103)	99 (96.1)	3 (2.9)	1 (1.0)	0 (0.0)	1.0 (1/103)
4b (*n* = 39)	31 (79.5)	2 (5.1)	4 (10.3)	2 (5.1)	15.4 (6/39)
4c (*n* = 25)	10 (40.0)	4 (16.0)	10 (40.0)	1 (4.0)	44.0 (11/25)
5 (*n* = 3)	0	0	2 (66.7)	1 (33.3)	100.0 (3/3)
Fisher Exact Test; p-value < 0.01*					
**Lesion morphology (n = 170)**					
Calcifications (*n* = 153)	126 (82.4)	8 (5.2)	16 (10.5)	3 (2.0)	
Asymmetric density (*n* = 5)	5 (100)	0	0	0	
Architectural distortion (*n* = 4)	2 (50.0)	1 (25.0)	1 (25.0)	0	
Spiculated mass (*n* = 8)	7 (87.5)	0	0	1 (12.5)	
**Calcification morphology (n = 153)**					
Coarse heterogenous (*n* = 55)	48 (87.3)	2 (3.6)	5 (9.1)	0	9.1 (5/55)
Amorphous (*n* = 38)	33 (86.8)	2 (5.3)	2 (5.3)	1 (2.6)	7.9 (3/38)
Fine pleomorphic (*n* = 52)	40 (76.9)	3 (5.8)	8 (15.4)	1 (1.9)	17.3 (9/52)
Fine linear (*n* = 8)	5 (62.5)	1 (12.5)	1 (12.5)	1 (12.5)	25.0 (2/8)
**Calcification distribution (n = 153)**					
Diffuse (*n* = 13)	11 (84.6)	1 (7.7)	1 (7.7)	0	7.7 (1/13)
Grouped (*n* = 76)	66 (86.8)	3 (3.9)	6 (7.9)	1 (1.3)	9.2 (7/76)
Regional (*n* = 30)	24 (80)	2 (6.7)	3 (10)	1 (3.3)	13.3 (4/30)
Linear (*n* = 15)	10 (66.7)	1 (6.7)	4 (26.7)	0	26.7 (4/15)
Segmental (*n* = 19)	15 (78.9)	1 (5.3)	2 (10.5)	1 (5.3)	15.8 (3/19)

* p-value of < 0.05 was applied as a threshold for statistical differences.

**Figure 2:**
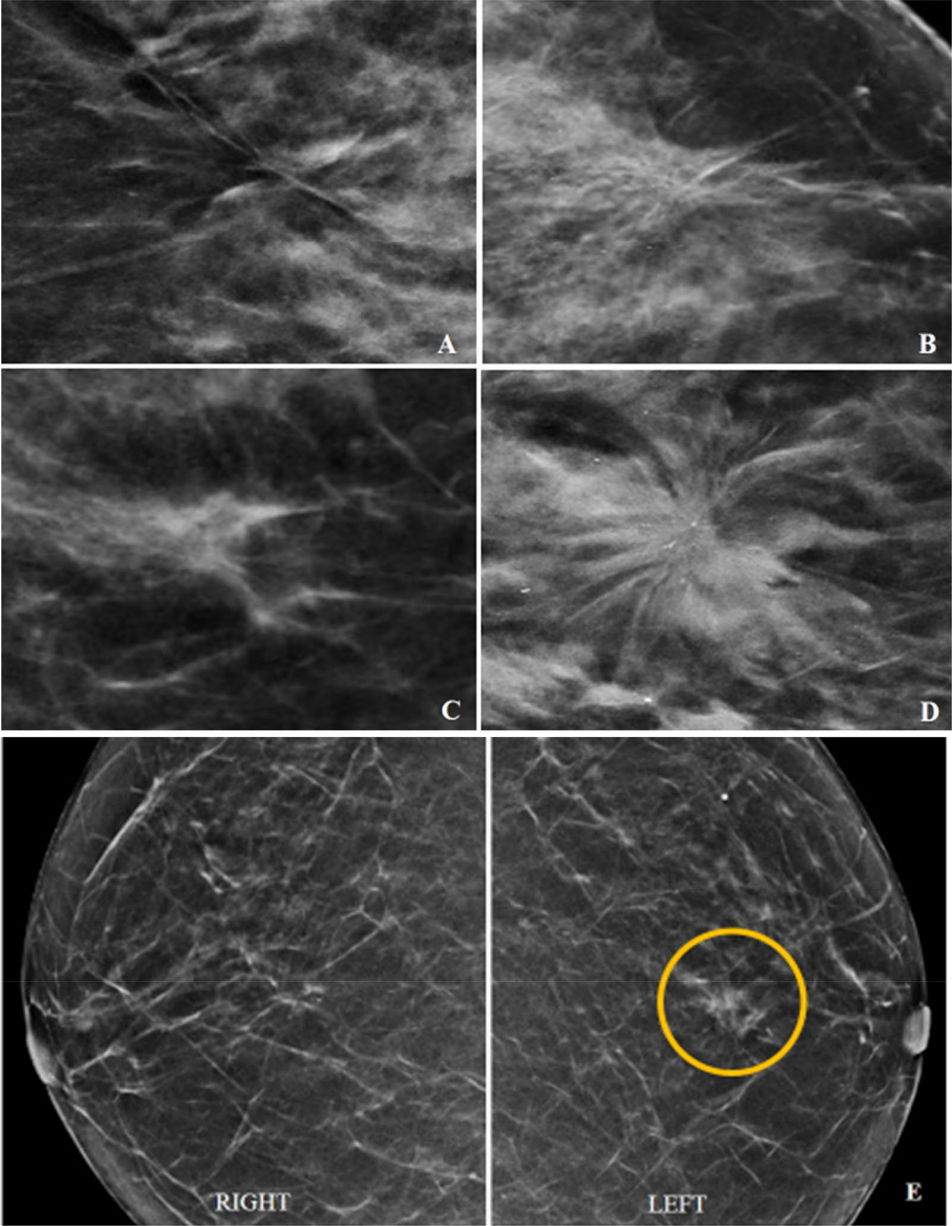
(**A–E**) showing architectural distortion (**A**,**B**) and spiculated density (**C**,**D**) with their respective HPE (benign vs malignant). Architectural distortion – no malignancy (**A**) and low-grade DCIS (**B**). Spiculated mass—periductal mastitis (**C**) and invasive ductal carcinoma (**D**). Figure **E** showing asymmetrical density (yellow circle) in the left breast in comparison with the right breast – no malignancy.

With regards to morphology, 87.6% calcifications and all asymmetric densities were benign. Malignancy were seen in 1 of 4 patients with architectural distortion and 1 of 8 patients with spiculated mass. Figure 2 demonstrates morphology of mass-like lesions on mammogram.

**Figure 3:**
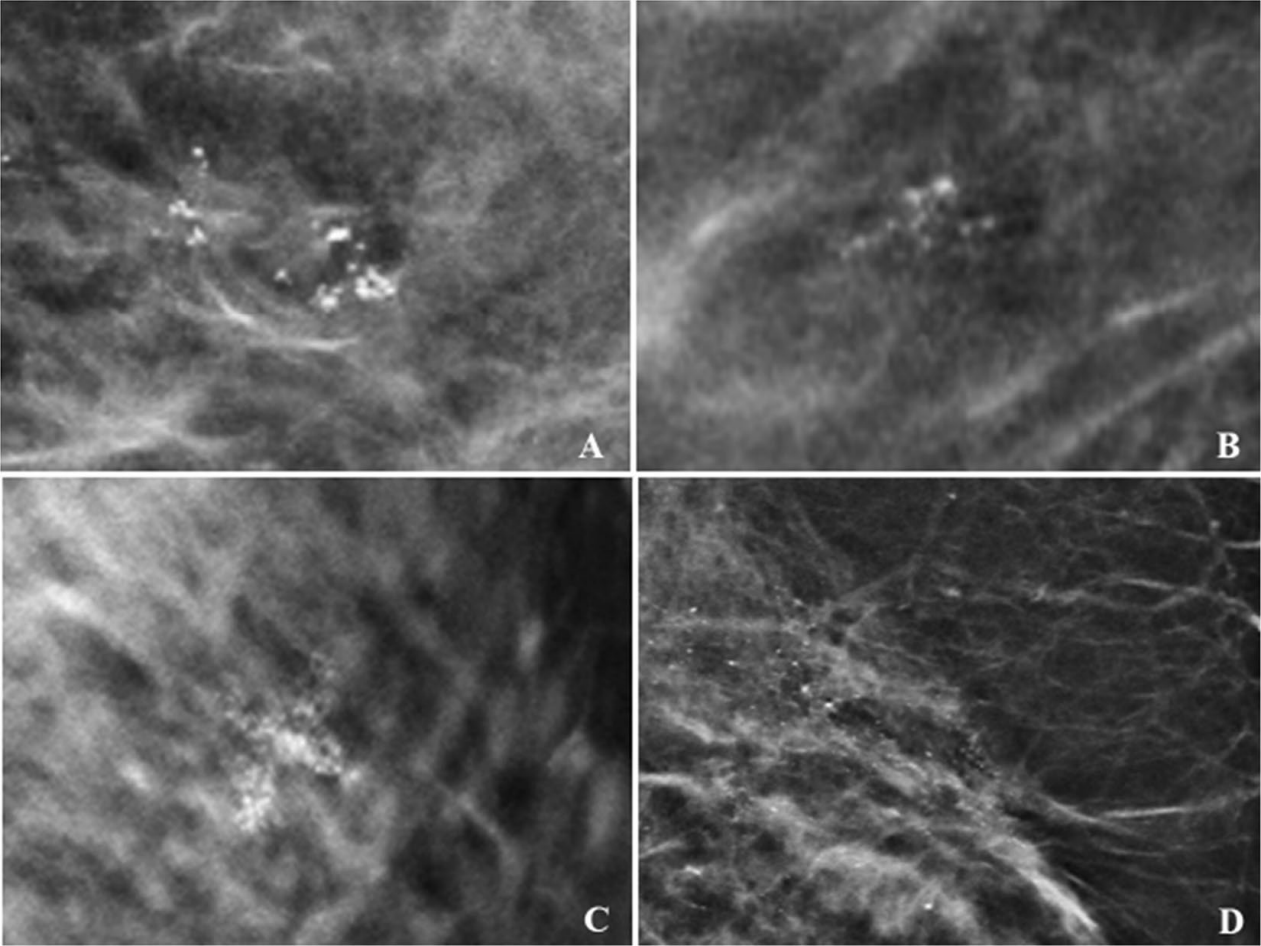
Calcifications morphology with benign histopathology on VABB. Example of coarse heterogenous in grouped distribution – fibrocystic (**A**). Amorphous in grouped distribution – no malignancy (**B**). Fine pleomorphic in regional distribution – no malignancy (after MDT discussion: discordant lesion) which revealed fibrocystic disease in surgical HPE (**C**). Fine linear in segmental distribution – UDH (after MDT: discordant lesion) which revealed fibrocystic disease in surgical HPE (**D**).

**Figure 4:**
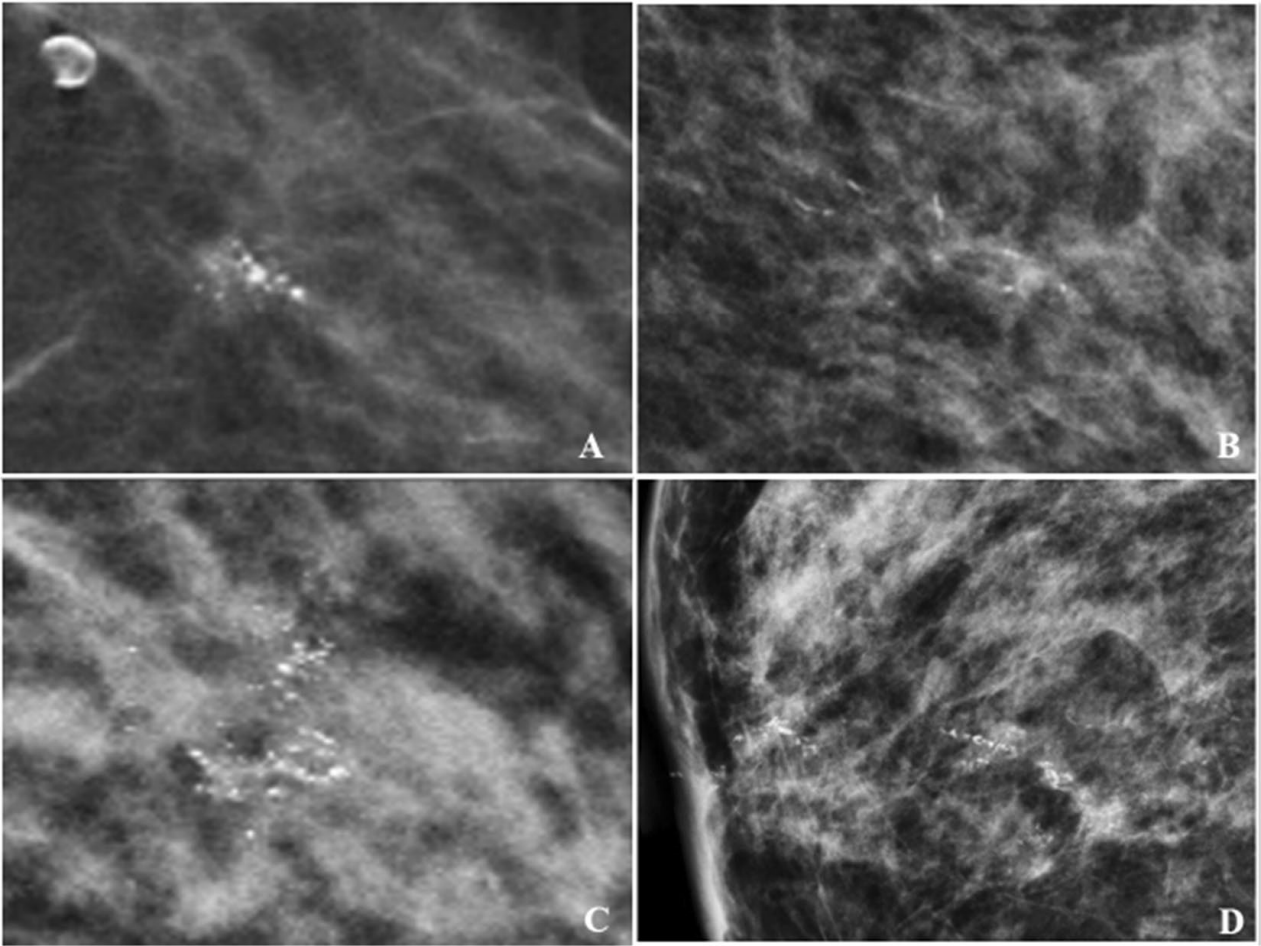
Calcifications morphology with malignant histopathology on VABB. Example of coarse heterogenous calcification in a grouped distribution – low-grade DCIS (**A**). Amorphous calcifications in segmental distribution – intermediate-grade DCIS (**B**). Fine pleomorphic calcifications in regional distribution – invasive ductal carcinoma (**C**). Fine linear in linear distribution – invasive ductal carcinoma (**D**).

For calcifications’ morphology, the probability of malignancy (PPV) was higher in fine linear (25%). For calcifications’ distribution, the probability of malignancy was 26.7% in linear category and 15.8% in segmental category. Figure 3 showed the examples of benign calcifications and Fig. 4 showed malignant calcifications.

### VABB HPE versus surgical HPE

We further explored the relationship between VABB and surgical HPE for 32 patients who underwent surgical excision due to malignant, borderline and imaging-pathology discordant lesions (Table 4). Underestimation of VABB was when there was an upgrade of benign to malignant, in-situ malignancy to invasive carcinoma and borderline lesions to in-situ malignancy or invasive carcinoma. Any upgrade from low-grade DCIS to moderate or high-grade DCIS and from moderate-grade DCIS to high-grade DCIS was also considered as an upgrade. In our study, diagnostic underestimation cases were only observed in ADH and high-grade DCIS.

**Table 4:** Comparison of VABB HPE and Surgical HPE.

VABB HPE	Surgical HPE						
	ADH	LG DCIS	IG DCIS	HG DCIS	IC	LCIS	Benign
ADH (*n* = 5)	3	1		1			
LCIS (*n* = 1)						1	
LG DCIS (*n* = 5)		5					
IG DCIS (*n* = 5)			5				
HG DCIS (*n* = 7)				6	1		
IC (*n* = 4)					4		
Discordant benign (*n* = 5)							5

* LG, IG, HG DCIS = Low, intermediate, high grade DCIS, IC = Invasive carcinoma.

**Figure 5:**
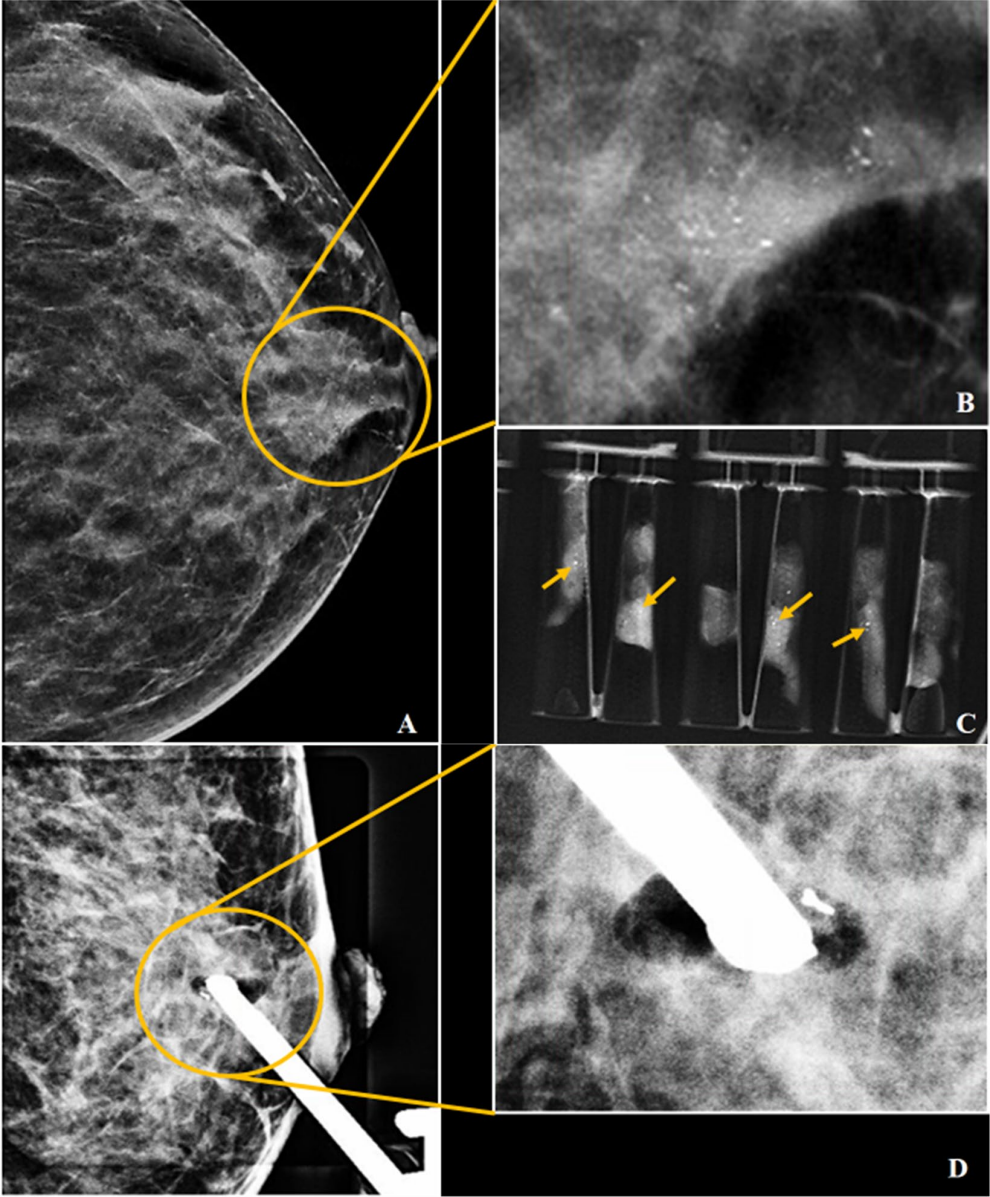
(**A–D**) Asymptomatic 42 year old lady who have strong family history of breast cancer came in for screening. The CC view of the left breast in C-view demonstrating fine pleomorphic calcifications with segmental distribution (yellow circle) in retroareolar region (**A**). Magnified view of area of calcifications as shown in (**B**). Specimens radiograph showing presence of microcalcifications within core tissues (yellow arrows in **C**). Post-VABB shows no residual calcifications with post-biopsy change and biopsy marker in situ (**D**). VABB HPE revealed ADH and was upgraded to low-grade DCIS in the subsequent surgery.

**Figure 6:**
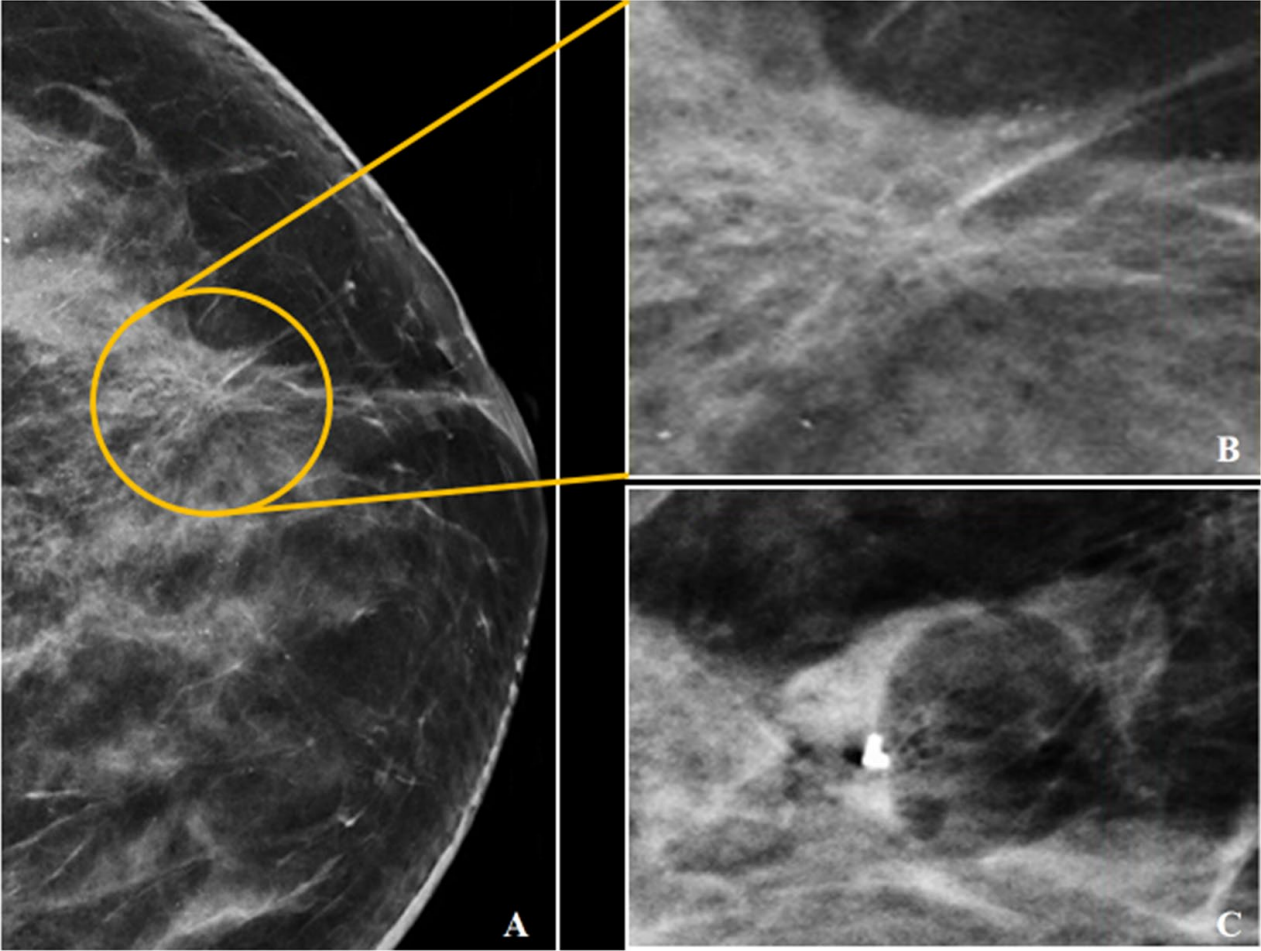
(**A–C**) A 42 year old lady with screen detected abnormality. The CC view of the left breast in C-view showed architectural distortion (yellow circle) in upper quadrant (**A**). Magnified view of region of interest as shown in (**B**). Post-VABB shows no residual lesion with resolved architectural distortion seen and post-biopsy change with biopsy marker in situ (**C**). VABB HPE revealed ADH and was upgraded to high-grade DCIS in the subsequent surgery.

There were 5 (3.6%) benign discordant lesions with no change in HPE after surgery. 6 out of 9 borderline lesions proceeded with surgery. 2 ADH were upgraded to DCIS representing 33.3% of diagnostic underestimation.The images of these cases were shown in Figs. 5 and 6. The remaining 3 borderline lesions did not proceed with surgery. 1 patient with FEA was lost to follow-up, 1 patient with ADH was not fit for surgery due to underlying advanced uterine carcinoma and MDT discussion justified surveillance only in the other ADH lesion in a 72 years old with low risk of carcinoma. No interval change was detected in a 48 months follow-up.

**Figure 7:**
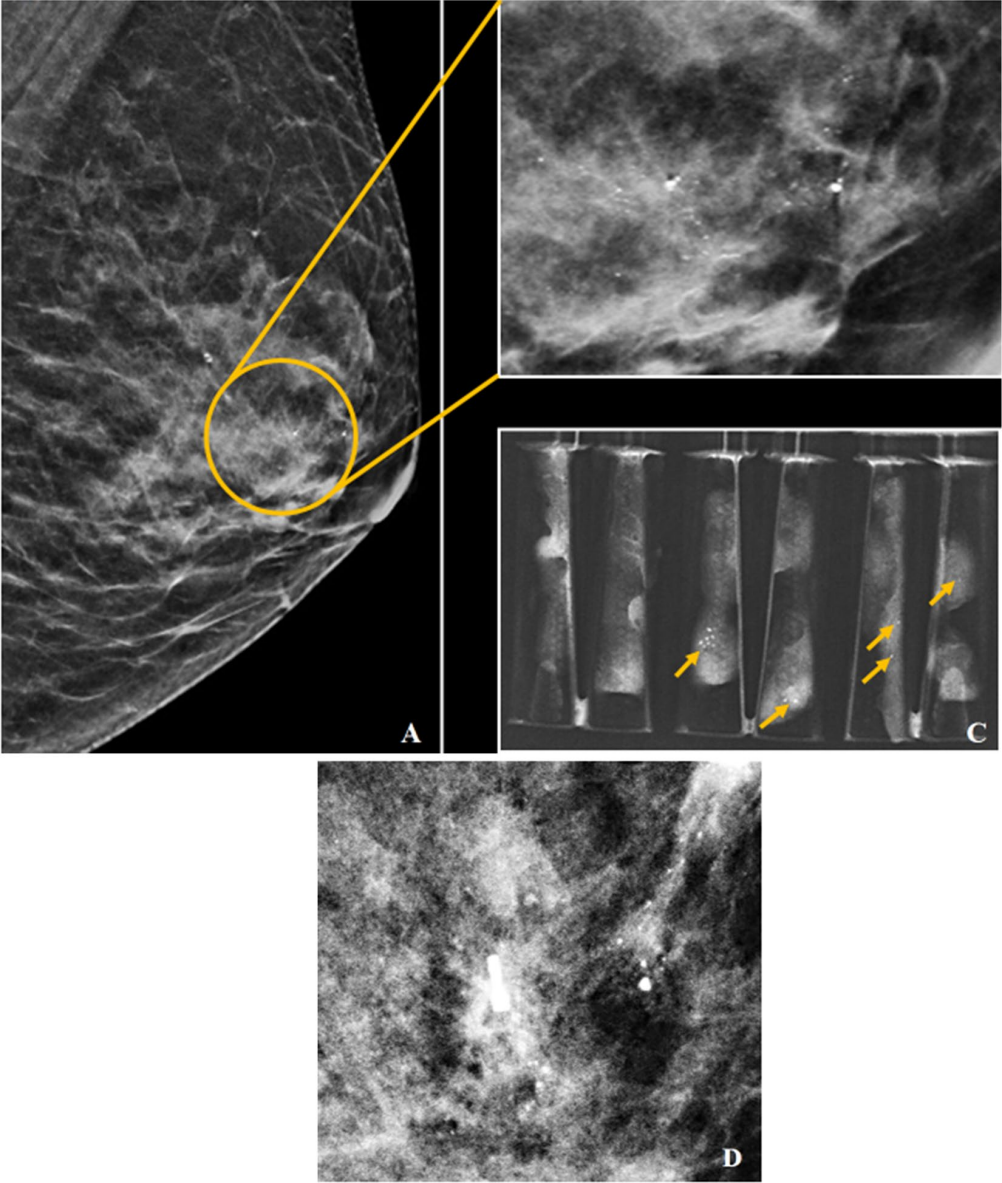
(**A-D**) A 67 year old lady with underlying contralateral breast carcinoma post mastectomy 16 years ago which came to our center for screening. MLO view of the left breast in C-view shows amorphous calcifications with segmental distribution (yellow circle) in retroareolar region (**A**). Magnified view of area of calcifications as shown in (**B**). Specimens radiograph showing presence of microcalcifications (yellow arrows in **C**). Post-VABB shows residual calcifications with post-biopsy change and biopsy marker in situ (**D**). VABB HPE revealed high-grade DCIS and was upgraded to invasive lobular carcinoma in the subsequent surgery.

In the malignant group, only one high-grade DCIS was upgraded to invasive lobular carcinoma at surgery representing 5.9% of diagnostic underestimation. The mammographic findings of this patient were illustrated in Fig. 7.

Overall, when comparing the VABB HPE with surgical HPE of the 32 patients, the underestimation rate was 9.4% (3/32), with 18.2% (2/11) false negative rate. Our study also revealed that VABB was specific (100%), sensitive (91.3%) and have high PPV (100%). in detecting malignant lesions. VABB also have high NPV (81.8%) in detecting benign lesions (Table 5).

**Table 5:** The statistical analysis of VABB HPE vs Surgical HPE.

VABB HPE	Final Result		Total	Predictive value	
	Malignant	Benign			
Malignant	21 (TP)	0 (FP)	21 (TP + FP)	PPV TP/(TP + FP)	100.0%
Benign	2 (FN)	9 (TN)	11 (FN + TN)	**NPV** TN/(FN + TN)	81.8%
**Total**	23 (TP + FN)	9 (FP + TN)	32		

Sensitivity TP/(TP + FN) = 91.3%.

Specificity TN/(FP + TN) = 100%.

## Discussion

Non-palpable mammographic lesions are one of the major challenges in diagnosis of breast cancer. Calcifications are the most frequent mammographic finding for early diagnosis of breast cancer^7,10–12^. In our study, we found that the distribution of malignancies in BI-RADS category is similar with the standard guideline by American College of Radiology^16^. Our results corroborate with the findings of other studies which showed that the likelihood of malignancy increases with BI-RADS category^7,11,12,17,18^.

Although calcifications play a crucial role in early breast cancer diagnosis, they are also associated with 63.2% of benign pathology^7^. The prediction of benign or malignant calcifications are possible by following ACR BI-RADS mammography lexicon guidelines. Previous study by Jun Liu et al. showed that the probability of malignancy in calcifications’ morphology was higher in fine pleomorphic (44.7%) while with respect to distribution, the highest percentage were in segmental distribution (78%) and 68% in linear distribution^7^. These findings were similar to our study.

Majority of previous literatures confirmed that VABB is a safe procedure, highly accurate technique and comparable to surgical biopsy for histopathological diagnosis in non-palpable breast lesions. A study performed by Penco et al. reported that the sensitivity of VABB ranged from 99.7% to 100% and false negative rate of 1.7% to 7.1% in 4086 patients within 10 years duration^19^. Tsai et al. reported that stereotactic VABB has a sensitivity of 95.24%, false-negative rate of 4.76% and NPV of 99.61% in 817 patients within 5 years duration^11^. Similarly, Kettritz et al.achieved sensitivity and NPV of more than 99%^17^. We postulate that our study produced a higher false-negative rate of 20% due to a smaller sample size (2/10) as compared to other studies. However, in line with previous literatures, we found that VABB shows high specificity (100.0%) and sensitivity (91.3%). We also proved that VABB has high PPV (100.00%) for malignancy and high NPV (81.8%) for benignity. This study also demonstrates that DBT-guided VABB is safe and reliable despite not having prior experience with the procedure.

As it is generally known, diagnostic underestimation and false-negative rate is higher in core needle biopsy (CNB) compared to VABB. According to previous literature, range of diagnostic underestimation by CNB for DCIS was from 27 to 59% and approximately 11.5% to 88% for ADH^13,14^. Unfortunately, stereotactic core needle biopsy is still the main choice for obtaining histological diagnosis in majority of hospitals in Malaysia due to cost. However, in the long run, VABB will reduce the need for surgery and reduce the incidence of delayed in diagnosis of cancer due to underestimation, which will then further burden the healthcare system.

We found that the DCIS and ADH underestimation rate of our VABB were in line with previous studies. In a previous study by Tsai et al.^11^, the DCIS underestimation rate was 16.7% while another study by Kettritz et al.^17^ revealed that underestimation of ADH and DCIS were 24% and 12%, respectively. Another recent study by Badan et al. reported the underestimation rate of ADH was 25% and DCIS was 14.28%^13^. According to Inyoung Youn et al., the underestimation rate of ADH was 33.3% on VABB although all microcalcifications were completely removed^20^. Our data confirmed the accuracy of VABB in detecting malignant lesions and we suggest for further surgical excision in borderline lesions for a more accurate diagnostic evaluation. This has become a normal practice in our centre. However, according to the international consensus for borderline lesions, surveillance instead of surgical excision is possible in older age groups since most of the invasive cancers that develop after ADH progress slowly^21^.

All benign lesions in our study were mostly followed-up until 48 months and were found to be true negative. This can obviate unnecessary operations for benign lesions diagnosed by VABB. The diagnostic accuracy does not depend on obtaining a large number of specimens, instead an adequate number of correctly targeted specimens is essential. Previous studies showed that even with standardized retrieval of 20 specimens per lesion, underestimation can still occur^22^. In our study, the number of specimens ranged from 5 to 27 specimens with mean of 6.8. Similarly, the mean number of core was 8.5 in previous study by Esen Gul et al. but they have proven that although the average core number was low, their false negative rate was 0% with the total excision rate of  almost 44%^22^.

The complication rate of stereotactic VABB is low, approximately 3.7%^18^. The commonest complication of stereotactic VABB was haematoma which was well-controlled by manual compression and none of the previous study had reported patient requiring any surgical intervention for management of the complications^7,12,18,23^. In our study, 98.8% of our patients had no complications and 1.2% have a small haematoma of less than 2 cm.

VABB is a well-tolerated procedure in our study. The majority of our patients only experienced mild pain (55.4%) and 25.9% of them did not experienced any pain during the procedure. Only 0.6% of the patients in our study complained of severe pain which was associated with larger sample size. This finding is in line with previous study conducted by Seely et al. which revealed an average pain score of 3.1 with stereotactic VABB which is in mild category^24^.

## Limitations

There are several limitations in our study. Firstly, due to the nature of the retrospective database at a single institution, some useful patient data were not well recorded. Our study has small sample size and may be insufficient in providing an accurate overall picture. Further clinical studies with multicenter collaborations may provide more accurate conclusion. Secondly, the benign lesions underestimation was mainly evaluated based on clinical follow-up at a maximum of 2 years duration in our study. A long-term follow-up is necessary for more accurate results.

## Conclusion

Our study has shown that DBT-VABB is reliable in the diagnosis of non-palpable breast lesions. DBT_VABB is also a tolerable procedure without major complication. It has high sensitivity, specificity, PPV and NPV in detecting malignant as well as benign lesions. Benign lesions diagnosed by VABB can be safely followed up without any surgical interventions and malignant diagnosis on HPE should undergo definitive surgical treatment. Subsequent surgical excision for all borderline lesion, especially ADH should be recommended regardless of complete removal of calcifications in VABB. Our study also confirmed that BI-RADS category assessment is effective to anticipate the probability of malignancy.

The promising results in our study supported the implementation of VABB as a standard tool for diagnosis of non-palpable mammographic lesions in all tertiary referral hospital.

## References

[1] Ar, M. O. H. P. I. K. N. Moh/p/ikn/01.16 (ar). *Malaysian Natl. Caner Regist. Rep. 2007–2011. Malaysian cancer Stat. data Fig.* **16**,

[2] O’Mahony, M. *et al.* Interventions for raising breast cancer awareness in women. *Cochrane Database Syst. Rev.* **2014**, (2014). 10.1002/14651858.CD011396.pub2 PMC6464597 28185268

[3] Coleman C (2017). Early detection and screening for breast cancer. Semin. Oncol. Nurs..

[4] Wilkinson L, Thomas V, Sharma N (2017). Microcalcification on mammography: Approaches to interpretation and biopsy. Br. J. Radiol..

[5] Clinical practice guidelines (CPG)-breast cancer. Academy of Medicine of Malaysia (2 Edition). (2018).

[6] Teh Y (2015). Opportunistic mammography screening provides effective detection rates in a limited resource healthcare system. BMC Cancer.

[7] Liu J, Huang L (2018). Image-guided vacuum-assisted breast biopsy in the diagnosis of breast microcalcifications. J. Int. Med. Res..

[8] Arancibia Hernández PL, Taub Estrada T, López Pizarro A, DíazCisternas ML, Sáez Tapia C (2016). Breast calcifications: description and classification according to BI-RADS 5th edition. Rev. Chil. Radiol..

[9] Yonekura R (2018). A diagnostic strategy for breast calcifications based on a long-term follow-up of 615 lesions. Jpn. J. Radiol..

[10] Allred DC (2010). Ductal carcinoma in situ: terminology, classification, and natural history. J. Natl. Cancer Inst. Monogr..

[11] Tsai, H., Fu, M. C., Hsu, O. J. K. J. & Chiu, M. H. H. Accuracy and outcomes of stereotactic vacuum-assisted breast biopsy for diagnosis and management of nonpalpable breast lesions. 1–6 (2019). doi:10.1002/kjm2.12100 10.1002/kjm2.12100 PMC11900702 31271510

[12] Safioleas PM (2017). The value of stereotactic vacuum assisted breast biopsy in the investigation of microcalcifications. A six-year experience with 853 patients. J. BUON.

[13] Badan GM (2016). Diagnostic underestimation of atypical ductal hyperplasia and ductal carcinoma in situ at percutaneous core needle and vacuum-assisted biopsies of the breast in a Brazilian reference institution. Radiol. Bras..

[14] Damnjanovic I (2015). Stereotactic vacuum-assisted breast biopsy: our experience and comparison with stereotactic automated needle biopsy. Bratisl. Lek. Listy.

[15] Lacambra MD (2012). Biopsy sampling of breast lesions: Comparison of core needle- and vacuum-assisted breast biopsies. Breast Cancer Res. Treat..

[16] D’Orsi CJ, Sickles EA, Mendelson EB, Morris EA, et al. ACR BI-RADS® Atlas, Breast Imaging Reporting and Data System. Reston, VA, A. C. of R. 2013. *No Title*.

[17] Kettritz U (2004). Stereotactic vacuum-assisted breast biopsy in 2874 patients: a multicenter study. Cancer.

[18] Girardi, M. T. V, Radiosenologia, S., Pederzoli, C. P., Baldo, V. M. & Garda, P. Stereotactic vacuum-assisted breast biopsy in 268 nonpalpable lesions Biopsia mammaria in stereotassi, vacuum-assisted, in 268 lesioni non palpabili. 65–75 (2008). doi:10.1007/s11547-008-0226-0 10.1007/s11547-008-0226-0 18338128

[19] Penco S (2010). Stereotactic vacuum-assisted breast biopsy is not a therapeutic procedure even when all mammographically found calcifications are removed: Analysis of 4,086 procedures. Am. J. Roentgenol..

[20] Youn I, Kim MJ, Moon HJ, Kim EK (2014). Absence of residual microcalcifications in atypical ductal hyperplasia diagnosed via stereotactic vacuum-assisted breast biopsy: is surgical excision obviated?. J. Breast Cancer.

[21] Rageth CJ (2019). Second International Consensus Conference on lesions of uncertain malignant potential in the breast (B3 lesions). Breast Cancer Res. Treat..

[22] Esen G (2016). Vacuum-assisted stereotactic breast biopsy in the diagnosis and management of suspicious microcalcifications. Diagnost. Interv. Radiol..

[23] Ariaratnam NS, Little ST, Whitley MA, Ferguson K (2018). Digital breast Tomosynthesis vacuum assisted biopsy for Tomosynthesis-detected Sonographically occult lesions. Clin. Imaging.

[24] Seely JM, Hill F, Peddle S, Lau J (2017). An evaluation of patient experience during percutaneous breast biopsy. Eur. Radiol..

